# Evaluator perceptions of NGO performance in disasters: meeting multiple institutional demands in humanitarian aid projects

**DOI:** 10.1111/disa.12419

**Published:** 2020-05-13

**Authors:** Liesbet Heyse, Fernando Nieto Morales, Rafael Wittek

**Affiliations:** ^1^ Associate Professor, Department of Sociology/ICS (Interuniversity Center for Social Science Theory and Methodology) University of Groningen Netherlands; ^2^ Associate Professor, Center for International Studies El Colegio de México Mexico; ^3^ Professor, Department of Sociology/ICS University of Groningen Netherlands

**Keywords:** governance structures, humanitarian aid, humanitarian crises, non‐governmental organisation (NGO), non‐profit organisation, organisational paradox, project performance

## Abstract

Providing aid in times of increasing humanitarian need, limited budgets, and mounting security risks is challenging. This paper explores in what organisational circumstances evaluators judge, positively and negatively, the performance of international non‐governmental organisations (INGOs) in response to disasters triggered by natural hazards. It assesses whether and how, as perceived by expert evaluators, CARE and Oxfam successfully met multiple institutional requirements concerning beneficiary needs and organisational demands. It utilises the Competing Values Framework to analyse evaluator statements about project performance and organisational control and flexibility issues, using seven CARE and four Oxfam evaluation reports from 2005–11. The reports are compared using fuzzy‐set Qualitative Comparative Analysis. The resulting configurations show that positive evaluations of an INGO's internal and external flexibility relate to satisfying beneficiary needs and organisational demands, whereas negative evaluations of external flexibility pertain to not meeting beneficiary needs and negative statements about internal control concerning not fulfilling organisational demands.

## Multiple institutional demands and performance in humanitarian INGOs

International non‐governmental organisations (INGOs) are important players in humanitarian crises. Until recently, their non‐profit character and voluntary base were assumed to make them more effective and efficient than market or state actors (Douglas, 1987; Hansmann, 1987). However, the performance of INGOs proved to be debatable, both in relation to complex emergencies (Sommer, 1994; Aall, Miltenberger, and Weiss, 2000) and disasters triggered by natural hazards (Tsunami Evaluation Coalition, 2006; Levine, Crosskey, and Abdinoor, 2011; Coyne, 2013). They experienced problems in adequately delivering aid to people in need, as was regularly reported in the *ALNAP Annual Review* of humanitarian action (see, for example, Borton and Robertson, 2002; Beck et al., 2003; Christoplos et al., 2004). Such disappointing performance, especially in the Great Lakes refugee crisis in the wake of the genocide in Rwanda in April 1994 (Millwood, 1996), led institutional and private donors, the media, and aid workers to call for increased accountability, transparency, and learning within the sector, which in turn generated standardisation and professionalisation processes (Barnett, 2013). Initiatives were launched to define shared minimum standards, such as the ‘Sphere Standards’ (Dufour et al., 2004), and compliance with harmonised reporting and evaluation mechanisms was demanded (Edwards and Hulme, 1996; Barnett, 2013). In addition, donors started to promote the ‘Logical Framework’ as a preferred approach for formulating project proposals and monitoring the implementation of aid. This linear and hierarchical format requires that projects are split up into goals, objectives (outcomes), outputs, and activities that are related to indicators, means of verification, and assumptions (Bakewell and Garbutt, 2005). Lastly, widespread insistence on the professionalisation of aid workers (Walker and Russ, 2010) generated more training efforts and attention to human resource management.

These ‘rationalisation’ and ‘projectification’ processes (Krause, 2014) reflect a trend towards managerialism in the sector (Roberts, Jones, III, and Fröhling, 2005; Hwang and Powell, 2009). This could be at odds with the exigencies of local crisis contexts (Edwards, 1999; Lindenberg and Bryant, 2001), requiring that INGOs operate according to predefined rules, standards, and procedures (such as the Logical Framework), whereas organisational action in such settings often needs to be flexible and event‐driven (Barnett, 2005). For instance, INGOs need to change their plans if rain suddenly floods an area after a period of long‐lasting drought, necessitating not only rescue and shelter but also interventions to prevent the outbreak of cholera and other waterborne diseases. Project managers and humanitarian aid workers need to be creative and adaptable, therefore, which implies ad hoc adjustments rather than adhering to predefined rules and procedures. Furthermore, standardisation and professionalisation can lead to risk‐averse behaviour in humanitarian INGOs (Barnett, 2013). Hence, pressures to align (headquarter and donor) accountability standards with flexibility requirements from the crisis zone can trigger intra‐organisational conflict and tensions (Edwards and Hulme, 1996a; Pache and Santos, 2010): if an organisation opts to remain flexible, it runs the risk of violating accountability principles and of potentially losing critical donor support. Conversely, standardised and supply‐driven aid responses may not always match beneficiary needs in a crisis area (Bradt, 2009; Darcy, 2009).

Humanitarian INGOs thus face multiple institutional demands (see, for example, Meyer and Rowan, 1977), that is, ‘various pressures for conformity exerted by institutional referents on organizations in a given field’ (Pache and Santos, 2010, p. 457). These pressures originate in different (sometimes opposing) regulatory regimes, normative orders, or logics (Kraatz and Block, 2008; Pache and Santos, 2010), and create two types of demands. First, ‘upward’ accountability, referring to standardisation pressures as they are frequently defined by donors and other external stakeholders (Edwards and Hulme, 1996a). Adhering to them meets organisational demands for survival (Edwards and Hulme, 1996a; Cooley and Ron, 2002; Barnett, 2013). Second, ‘downward accountability', signifying pressures for flexible operations to meet beneficiary needs (Edwards and Hulme, 1996a). The core dilemma of INGOs is that the two types of institutional demands often are in competition, but both are central to performance.

It is asserted regularly that humanitarian organisations ‘follow the money’ and therefore focus more on establishing ‘upward accountability’ to their donors (by means of standardisation) than on ‘downward accountability’ (by acting flexibly) to their beneficiaries (Edwards and Hulme, 1996a), since the latter have much less power as compared to the former (Christensen and Ebrahim, 2006). This paper assesses empirically to what degree humanitarian INGOs are able to meet *both* organisational demands and beneficiary needs. It analyses 11 evaluation reports of humanitarian aid projects of CARE (Cooperative for Assistance and Relief Everywhere) and Oxfam (Oxford Committee for Famine Relief) during the period 2005–10. To increase comparability, the spotlight is on one specific intervention milieu: disasters triggered by natural hazards.

The theoretical point of departure is the ‘Competing Values Framework’ (CVF) (Quinn and Rohrbaugh, 1981, 1983; Cameron and Quinn, 2011; see also Smith and Lewis, 2014), which explains how apparently contradictory demands can be met (Denison, Hooijberg, and Quinn, 1995), building on the literature on paradox (Smith and Lewis, 2014) and ambidexterity (Raisch and Birkinshaw, 2008). The argument is that organisations can meet multiple demands simultaneously if they can embrace the multiplicity (or paradox).

This paper adds to extant research in three ways. First, there is little systematic, comparative empirical work on non‐profit performance and effectiveness in general (Lecy, Schmitz, and Swedlund, 2012), and specifically, on humanitarian aid and project management; this study contributes to the closing of this gap. Second, it administers a configurational approach to performance (Ragin, 1989, 2008; Meyer, Tsui, and Hinings, 1993) by applying the CVF to the performance of INGOs in humanitarian aid projects, allowing one to gauge the relative importance of different organisational conditions for (in)adequate performance in the sector. Third, it scrutinises statements by evaluators about the project performance of INGOs in relation to disasters triggered by natural hazards and their views on the organisational conditions pertaining to performance. Evaluations can be considered as expert judgements, which so far have been hardly employed in academic research. Fuzzy‐set Qualitative Comparative Analysis (fsQCA) was used to gain insight into general patterns in the coded evaluation reports.

The next section presents the theoretical framework of this study. It is followed by the data and methods, the results of the fsQCA, and an analysis of particular cases. The final section contains some conclusions.

## The CVF and performance in humanitarian organisations

The CVF (Quinn and Rohrbaugh, 1981, 1983; Cameron and Quinn, 2011) structures a variety of theoretical perspectives outlining different determinants of organisational effectiveness and performance. This approach is utilised here to theorise about INGO (project) performance in humanitarian crises in terms of competing institutional demands and potential management tensions. Given that the study does not employ statistical methods, formal hypotheses are not formulated. Instead, general expectations are derived from the theory (see Figure 2 for a summary).

**Figure 1:**
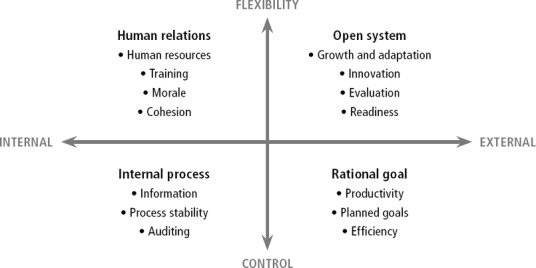
The CVF **Source**: authors, adapted from Quinn and Rohrbaugh (1983).

Two dimensions of the CVF are of particular interest to the current study (Quinn and Rohrbaugh, 1981, 1983; Cameron and Quin, 2011): (i) the degree to which effectiveness requires either control or flexibility; and (ii) an internal–organisational (such as staff or procedures) versus an external focus (such as relations with stakeholders). Cross‐classification produces four ideal–typical models of organisational performance (see Figure 1).

The control–flexibility dimension reflects the challenges of humanitarian INGOs, which juggle flexibly meeting beneficiary needs while simultaneously keeping control to meet organisational demands. According to previous research, organisational responses to crises can range between flexible/emergent and established/structured responses, or a mixture of the two (Brouilette and Quarantelli, 1971; Dynes and Aguirre, 1979). Humanitarian INGOs also need to pay attention to the internal–external dimension, since meeting organisational demands (such as donors’ expectations) not only necessitates internal control but also external accountability, whereas meeting beneficiaries’ needs requires consideration of local circumstances and (internal) response capacity.

The four ideal–typical models of organisational performance have been used to formulate competing theoretical expectations to answer the question as to under which organisational and contextual conditions good performance is assured (see, for example, Smith and Lewis, 2014). This study applies the CVF in relation to a multiple institutional demands perspective (Pache and Santos, 2010). It assumes that specific institutional demands may be more or less salient, and more or less compatible (see, for example, Besharov and Smith, 2014), and that each of the four ideal–typical models captures a specific combination of this salience and compatibility. Hence, to achieve good performance in terms of meeting beneficiary needs and organisational demands, INGOs need to be able to operate simultaneously within a flexible and control model. The remainder of this section presents the underlying theoretical reasoning and applies it to the humanitarian context.

### Meeting beneficiary needs

Internal and external flexibility is key to fulfilling beneficiary needs (see the upper quadrants of Figure 1). To perform well, therefore, it is assumed that organisations need to pay attention internally to human resources, morale, cohesion, and training—as reflected in the human relations model (HRM)—as well as to adjust to changing circumstances and to ensure external support—as reflected in the open systems model (Quinn and Rohrbaugh, 1983, 1981).

Internally, a strong focus on the HRM can help to create a cohesive and committed workforce. Fostering motivation and commitment while investing in staff through training and development (Panayotopoulou, Bourantas, and Papalexandris, 2003) is assumed to lead to higher quality and tailor‐made service provision, and thus to high overall organisational performance (Cameron and Quinn, 2011; Hartnell, Ou, and Kinicki, 2011). In the humanitarian setting this means that it is not only necessary that resources (staff included) can be made available and deployed quickly (Houghton and Emmens, 2007), but also to hire professional, well‐trained, and skilled aid workers who are knowledgeable about the local context. For instance, aid is less likely to meet beneficiary needs if personnel are not aware that, if placed in unsafe locations, latrines might not be utilised by women and so waterborne diseases can persist or worsen.

Regarding external flexibility, good organisational performance is assumed to require the ability to achieve congruence with and to adapt to changes in the environment (Buenger et al., 1996). This entails risk‐taking and creativity (Cameron and Quinn, 2011; Hartnell, Ou, and Kinicki, 2011). Apropos of humanitarian aid, this means responding to the circumstances of the local crisis context, which is accomplished by ensuring the participation of and ownership by local communities and governments, as well as good communication and coordination with them (Byrne, 2003; UN OCHA, 2012). For instance, not consulting with local actors before installing water pumps after an earthquake might result in them being left unused, because they may not meet local needs. Moreover, INGOs commonly operate in politically‐sensitive and conflict‐ridden settings that need to be monitored and managed. A case in point is that aid might be diverted to particular groups. INGOs thus need to strike a balance between being responsive to their environment without being captured by it (see, for example, Weiss and Collins, 2018), otherwise aid may not meet beneficiary needs.

### Satisfying organisational demands

To meet organisational demands effectively—such as those pertaining to accountability, resources, and survival—it is assumed that organisations need to be in control of their operations by externally securing resource endowments and internally achieving consistency and predictability of activities (Quinn and Rohrbaugh, 1981, 1983; Cameron and Quinn, 2011). Recent scandals involving fraud and sexual abuse within several INGOs indicate that organisations are not always in control of their operations and staff, which can seriously harm the reputation of the organisation and beneficiaries, as well as threaten their survival.^1^

The CVF's internal process and rational goal models reflect the importance of internal and external control for effective organisational performance. The *external control dimension* relates to an organisation's need to survive in a competitive environment, requiring the acquisition of sufficient resources and demonstration of being a serious competitor that can deliver (Cameron and Quinn, 2011; Hartnell, Ou, and Kinicki, 2011). Consequently, managing relationships with stakeholders (especially donors in the case of humanitarian INGOs) and upholding legitimacy are crucial to organisational survival (Buenger et al., 1996; Panayotopoulou, Bourantas, and Papalexandris, 2003), underlining the need for public relations (PR) and accountability mechanisms.

The humanitarian sector can be regarded as a competitive market, since aid INGOs compete for and are dependent on the grants of institutional donors and donations from private entities (Barnett, 2013). Institutional donors in particular (including governments and international organisations such as the European Union (EU) and the United Nations) require that INGOs follow strict formal procedures regarding transparency and accountability, and partly base their funding decisions on compliance with them. Hence, aid INGOs need to manage their reputation, as well as donor relationships, to survive and secure funding (Barnett, 2013).

The *internal control dimension*, meanwhile, assumes the importance of being in charge of organisational processes by means of hierarchy, standard operating procedures, and oversight (Buenger et al., 1996; Cameron and Lavine, 2006). Setting clear goals and expectations for employees and departments and monitoring progress and performance (Panayotopoulou, Bourantas, and Papalexandris, 2003) establish focus in the organisation, which harnesses consistency and efficiency in operations and contributes to goal achievement (Hartnell, Ou, and Kinicki, 2011; Cameron and Lavine, 2006). Control requires well‐functioning information systems (Quinn and Rohrbaugh, 1981, 1983; Panayotopoulou, Bourantas, and Papalexandris, 2003), next to effective planning and financial management systems.

Humanitarian INGOs need to make sure, therefore, that project implementation stays in line with strategy, mission, and values and with sectorial standards and donor demands. This holds especially for humanitarian aid INGOs funded by institutional donors to which they have submitted project proposals with a detailed outline of a time frame and activities using the Logical Framework (Roberts, Jones, III, and Fröhling, 2005).

It is important to emphasise that INGOs aim to fulfil their goals in very complex environments, characterised inter alia by politics, different organisational interests, and a lack of information and coordination (Heyse, 2007). In such settings, the achievement of goals in the instrumental manner described above is challenging, posing the question as to whether the requirement of internal control as formulated in the CVF also applies to humanitarian INGOs.

**Figure 2:**
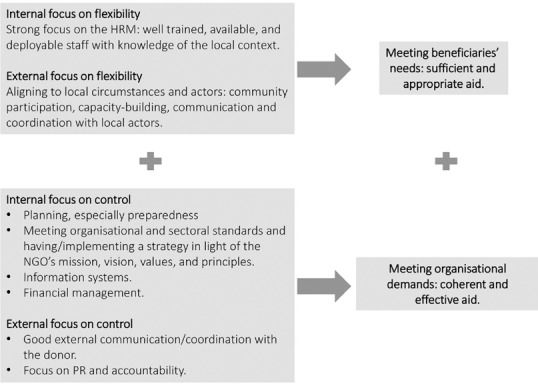
The theoretical framework **Source**: authors.

Figure 2 summarises the study's theoretical expectations derived from the CVF. Internal and external flexibility in particular is expected to be important for successfully meeting beneficiary needs, and internal and external control for satisfying organisational demands.

## Method

Text analysis was applied to seven evaluation reports on the project performance of CARE and four evaluation reports of Oxfam. The use of evaluation reports allows one to extract and scrutinise the expert judgements of evaluators who have been assigned to assess a certain project or particular projects.

Text analysis is useful to gain access to ‘the world of meanings, values, and norms’ as found in the content of communication (Popping, 2000, p. 1). The current study aimed to discover the representational interpretations of the authors of these reports, since its interest is the intended meaning of the evaluators (Shapiro, 1997, in Roberts, 2000, p. 262). Simultaneously, it sought to uncover via instrumental text analysis individual, societal, or other characteristics of which the producers and the readers of the documents might be unaware (Roberts, 2000, p. 262). As a result, the producers of the reports were treated as *informants*.

The investigation is based on a specific kind of organisational discourse that should be handled with care. For instance, evaluations are produced in a political environment in which reputations and money are at stake, possibly leading to negotiations about the contents (see, for example, Carlsson, Köhlin, and Ekbom, 1994). Funding pressures and media exposure might make aid organisations risk averse and not eager to be open about their mistakes and errors (Smillie, 2012). This might produce bias in publicly available evaluations—although, in practice, many of them are not disseminated or shared with a larger audience (Vaux, 2006). Furthermore, the quality of evaluations in the humanitarian sector has been criticised (Forss et al., 2008), which is addressed by selecting evaluations *led by external evaluators* ^2^ because these are assumed to be more robust as compared to internal evaluations (Afek‐Eitam and Ferf, 2015; Buchanan‐Smith, Cosgrave, and Warner, 2016).

### Unit of analysis and report selection

The study's unit of analysis is a report on an aid project (or projects) implemented by an INGO following a disaster triggered by a natural hazard in a particular place and at a particular time. The outcome of interest is the *project performance* of the INGO— that is, meeting beneficiary needs, organisational demands, or both. End‐of‐project evaluations were appraised because the point of interest is assessment of the project *after* the completion of activities. End‐of‐project evaluations focus on gauging completed projects and thus contain statements about outcomes *and* performance following finalisation, as opposed to mid‐term reviews or real‐time evaluations. Moreover, the former are more often performed by external evaluators than is the case with the latter (Afek‐Eitam and Ferf, 2015).

Since the spotlight is on the relationship between organisational circumstances and INGO performance in humanitarian projects, as perceived by evaluation experts, it is desirable to control contextual variation in the selected reports as much as possible (Yin, 2013). One distinct type of humanitarian emergency was chosen, therefore, instead of multiple varieties: disasters triggered by natural hazards (such as earthquakes, floods, and storms). Humanitarian responses to such events generally involve assisting local authorities with supplying or directly providing basic necessities (food, hygiene, medicine, shelter, and water) and facilitating the first steps towards regaining livelihoods (see, for example, Stokke, 2007). Studying INGOs in this one context ensured that the general characteristics of this particular type of humanitarian emergency remained relatively stable. In addition, evaluation reports on projects implemented by INGOs in a particular time frame (2005–10) were selected, so that the organisational background of the organisation can be assumed to be relatively stable. Lastly, the decision was taken to concentrate the analysis on two INGOs that are quite similar in size and approach: CARE and Oxfam are sizeable and well‐known organisations that work on development and humanitarian issues, preferably through local communities and partners. These similarities helped in better ‘isolating’ the specific role of the particular CARE and Oxfam projects and the teams implementing them, which is the focus of the analysis. Thus, variation in performance is more likely to relate to the specifics of the teams and projects, rather than to the overall characteristics of the organisations, the crisis context, or the period under review.

### Report selection and cases

CARE is one of the largest humanitarian INGOs in the world. Its work centres on fighting global poverty, through development and relief initiatives, often via local partners. CARE is a global confederation, composed of 14 national members, with an international secretariat in Geneva, Switzerland, which also hosts the Global Emergency Group. Taking 2010 as a point of reference, CARE spent more than USD 655 million in 87 countries as part of 905 development and humanitarian aid projects (CARE International, 2010). Almost 61.5 per cent of its funding base comprises contributions from institutional donors such as national governments and the EU (CARE International, 2010).

CARE is known for its open approach to evaluation: it publishes many of its project reports online (almost 1,700 as of this writing, May 2019), as well as within the Humanitarian Evaluation and Learning Portal (HELP) of the Active Learning Network for Accountability and Performance in Humanitarian Action (ALNAP). CARE participated in the Humanitarian Genome (HG) Project,^3^ a Google‐style search engine containing the evaluation reports of this and other INGOs, such as Oxfam, Save the Children, and World Vision.

For practical reasons, only reports in English were selected from the CARE evaluation database.^4^ Of the 43 reports in English on humanitarian aid projects in this database from between 2005 and 2010, 33 were external end‐of‐project evaluations, of which 28 were single agency reports (that is, no joint or multi‐agency appraisals). Of these 28, nine fell into the category of disasters triggered by natural hazards:

- *Independent Evaluation of CARE's Humanitarian Response to Flooding Resulting from Tropical Storm Jeanne in Haiti (North‐west and Artibonite Provinces)* (2005);^5^
- *Independent Evaluation of CARE International's Earthquake Response in Northern Pakistan* (2006);^6^
- *Emergency Response to Disaster Affected Population (ERDAP) Project – Final Evaluation Report* (Kenya, floods) (2007);^7^
- *Final Evaluation of CARE Australia Supported Tsunami Response in Trincomalee and Batticaloa Districts of Sri Lanka* (2007);^8^
- *Emergency Flood Response Evaluation (October 2007 to January 2008)* (2008);^9^
- *Independent Evaluation of CARE Bangladesh's Cyclone Sidr Response Program* (2008);^10^
- *Evaluation of CARE Myanmar's Cyclone Nargis Response* (2008);^11^
- *External Evaluation of the Response Actions implemented by CARE Central America Nicaragua during the Emergencies: Hurricane Felix and the Flooding of the Río Grande in Matagalpa* (2008);^12^ and
- *Pakistan Floods 2010: Evaluation of CARE's DEC Phase 1 and DFID Dadu Projects* (2010).^13^

Oxfam, meanwhile, is an international confederation of 17 organisations working together with partners and local communities to reduce poverty and injustice. The confederation has a secretariat in Oxford, United Kingdom, and was established in 1995, although the name dates back to the Second World War. Oxfam implements rights‐based sustainable development programmes and engages in public education, campaigns, advocacy, and humanitarian assistance. In 2010, it spent EUR 660 million. Almost 40 per cent of its funding comes from national governments and EU institutions (Oxfam, 2010).

The reports of Oxfam were selected with the assistance of HELP. Twenty‐nine Oxfam reports related to disasters triggered by natural hazards were identified in the time frame 2005–11. Only five of them were single agency end‐of‐project evaluations. All of them are publicly available:

- *Evaluation of the Bangladesh Emergency Flood Response* (2005);^14^
- *Evaluation of Humanitarian Response to Floods in the San Julian Municipality, Santa Cruz, Bolivia* (2006);^15^
- *Georgia Flood Response Programme, Khulo District, Republic of Georgia, April–December 2005* (2006);^16^
- *Evaluation of the Response to Hurricane Dean in Jamaica, St. Lucia and Dominica* (2008);^17^ and
- *Evaluation Report – Typhoon Ketsana Emergency Response Project in Kon Tum Province – RVNA79* (2011).^18^

### Coding of reports

Inputs for the coding scheme were derived from the HG,^19^ an advanced search engine consisting of 270 codes that was developed, tested, and employed by a team of five coders from 2011–13.^20^ For this study, it was indicated per code whether a statement is positive, negative, or neutral. The first and the second authors independently coded a subsample of reports and discussed the results. Since there was little substantive variation in the coding, the first author proceeded with coding each of the selected reports from beginning to end.

Each time a statement was made in the body text of the report about organisational demands, beneficiary needs, or organisational factors relating to internal and external flexibility and control, as operationalised in the coding book (see the annexe), the statement was coded appropriately. If it was clear that a statement was negative or positive in tone, this was also specified. A text fragment could be assigned multiple codes, resulting in a code being allotted 3,591 times.

The CARE report on Cyclone Sidr in Bangladesh in 2008 received most coding (550) and the Oxfam report on the emergency flood response in Bangladesh in 2005 received the least coding (190). Overall, there was quite a balance in the percentage of negative and positive statements coded per report in the sample of 14 reports, demonstrating that evaluators were fairly even‐handed in addressing strong and weak points in the projects under review. The only exception was the Oxfam report on the humanitarian response to the floods in San Julian Municipality, Santa Cruz, Bolivia, in 2006, in which case many more positive statements on project performance and organisational issues were coded than were negative statements. This potential bias led to the decision to drop this report from the subsequent analysis. Furthermore, coding revealed that CARE reports on the 2007 and 2008 flood responses in Kenya and Uganda, respectively, were not suitable for the analysis because they lacked statements on multiple dimensions of interest to the study, including coherence, capacity‐building, staff deployments, and training. Given that there were no other evaluation reports on the activities of these two INGOs with respect to disasters triggered by natural hazards in the chosen time frame, the study continued with the 11 other reports (CARE: seven; Oxfam: four).

### Operationalisation and measurements

In terms of *performance*, the study was interested in the evaluator's judgement of to what extent CARE and Oxfam were able to meet both beneficiary needs and organisational demands as part of a given project. The operationalisation of these two variables was based on the Organisation for Economic Co‐operation and Development–Development Assistance Committee (OECD–DAC)'s criteria for evaluations, that is, appropriateness and coverage as criteria reflecting beneficiary demands, and coherence and effectiveness as criteria reflecting organisational demands:^21^

- **Beneficiary needs.** The study coded evaluator statements pertaining to two well‐known and widely used (OECD–DAC) criteria in the sector:
  (i) *Appropriateness beneficiaries*—the extent to which ‘the aid activity is suited to the priorities and policies of the target group, recipient’ (Chianca, 2007, p. 43). An example quotation (negative statement) is: ‘Seeds were distributed to tide over the loss in next season, however this was not too helpful to those who did not own land or had lost their lands in these floods. Need based intervention could have been more helpful in responding to people's specific needs’ (Oxfam, *Evaluation of the Bangladesh Emergency Flood Response*, p. 12).
  (ii) *Coverage*—this concerns who was supported and reached by humanitarian action, and who was not (and whether the groups with most needs were reached). An example quotation (positive statement) is: ‘The coverage was as good as could be achieved taking into account accessibility and CARE‐P sought in difficult circumstances to access remote areas—the use of helicopters was critical in this respect’ (CARE, *Independent Evaluation of CARE International's Earthquake Response in Northern Pakistan*, p. 31).^22^
- **Organisational demands.** The study coded evaluator statements pertaining to two well‐known and widely used (OECD–DAC) criteria in the sector:
  (i) *Coherence*—the extent to which organisational policies and actions are consistent, and all policies take into account humanitarian and human rights considerations. An example quotation (negative statement) is: ‘CARE's treatment of gender was mixed. On the one hand, gender analysis informed the targeting of food distribution while the integration of gender considerations in the programming appears to have been on an ad hoc basis. While CARE staff have received some level of gender awareness training, no policy, strategy or guidelines appear to be in place to promote the pursuit of gender equity’ (CARE, *Independent Evaluation of CARE's Humanitarian Response to Flooding Resulting from Tropical Storm Jeanne in Haiti*, p. 20).
  (ii) *Effectiveness*—this reflects the extent to which the activity/programme achieved its stated purpose in time, or whether this can be expected to happen on the basis of the outputs, in time. This code also included text on cost effectiveness. An example quotation (positive statement) is: ‘the achievement of 88km of repaired canal through cash for work implementation implies a higher achievement of benefited farmers than the set target: a rough estimation could show at least 2,450 households benefited because there was a total of 49 hamlets which benefited from repaired irrigation canal, and each hamlet should have at least 50 households’ (Oxfam, *Evaluation Report – Typhoon Ketsana Emergency Response Project in Kon Tum Province*, p. 13).

In terms of *organisational conditions* leading to performance, the study coded the evaluator's statements about the organisation's flexibility and control, both internally and externally, as follows:

- **Internal flexibility**—whether or not there was a strong internal focus on the HRM was captured by coding the evaluation reports in terms of positive, neutral, and negative statements related to three factors: (i) how quickly and appropriately staff were deployed; (ii) the training of staff; and (iii) staff knowledge of the local context. An example quotation (positive statement) is: ‘On the day of the Tsunami, CARE mobilized its staff from development programs and deployed them to the worst‐affected areas in the north, east and Hambantota in the south’ (CARE, *Final Evaluation of CARE Australia Supported Tsunami Response in Trincomalee and Batticaloa Districts of Sri Lanka*, p. 10).
- **External flexibility**—whether or not there was a strong external focus on local circumstances was gauged by coding the evaluation reports in terms of positive, neutral, and negative statements related to three factors: (i) local capacity‐building activities by the organisation; (ii) community‐based activities (allowing participation, creating ownership); and (iii) external communication and coordination with local actors, such as governments, community groups, and local partners. An example quotation (negative statement) is: ‘Regarding the decision making process, and reporting on the achievements made by the project, there is little or no evidence that the beneficiaries participated in such processes. Based on the discussions held with Oxfam HPO [humanitarian programme officer], GRCS [Georgia Red Cross Society] in Batumi, the government officials in Khulo, and the contractor (“PONI LTD”), it would seem that all the key decisions were taken between these “four key groups”’ (Oxfam, *Georgia Flood Response Programme*, p. 31).^23^
- **Internal control**—whether or not a given project had a strong internal focus on control was determined by coding the evaluation reports in terms of positive, neutral, and negative statements related to four factors: (i) planning; (ii) meeting organisational or sectorial standards as well as having/following a strategy; (iii) information systems; and (iv) financial management. An example quotation (negative statement) is: ‘Given the lack of preparedness and human resource capacity within the organisation, responding to the humanitarian imperative was difficult and risky. It is likely that this unpalatable choice—between responsibility for existing beneficiaries and for those affected by the cyclone—contributed to the caution and delayed decisions which hampered operations in phase 1’ (CARE, *Evaluation of CARE Myanmar's Cyclone Nargis Response*, p. 30). An example quotation (positive statement) is: ‘expenditure on the whole is as planned, with moderately higher actual expenditure on; Oxfam logistics costs; the sewerage system: and the water system. On the other hand, actual expenditure was moderately lower on; GRCS logistics costs; Oxfam HR costs; and GRCS HR costs. Actual expenditure on the software elements, workshops and publications was as planned’ (Oxfam, *Georgia Flood Response Programme*, p. 16).
- **External control**—whether or not the organisation had a strong external focus on control was ascertained by coding the evaluation reports in terms of positive, neutral, and negative statements related to two factors: (i) PR and accountability activities or systems; and (ii) external communication and coordination with donors. An example quotation (negative statement) is: ‘Actually, what would have been useful was a complaints mechanism for partners to feedback to Oxfam. While some complaints could have gone to the in‐country team, the issues around management should have gone up the line: a focal point in the Barbados office would have been useful’ (Oxfam, *Evaluation of the Response to Hurricane Dean in Jamaica, St. Lucia and Dominica*, p. 19).

## Analysis

Rather than providing a single recipe for performance, the CVF implies that INGOs need to balance diverse combinations of factors to meet both beneficiary needs and organisational demands. This suggests that one formula may not exist, and that positive or negative performance may vary in different projects and circumstances. A method that is particularly useful for evaluating the empirical validity of alternative mixtures of conditions ('recipes') in small‐N samples is Qualitative Comparative Analysis (QCA) (Mahoney and Goertz, 2006; Ragin, 2008; Thiem, Spöhel, and Duşa, 2016), which permits a comparison of conditions and their relations as if they were sets. Set logic and Boolean operations formally compare subset relations to reveal consistent patterns of co‐occurrence, including whether fulfilling beneficiary demands is consistently related to external flexibility in the studied reports. Furthermore, fsQCA, as opposed to ‘crisp‐set analysis', is employed, meaning that variables vary between zero and one (these are called ‘fuzzy scores'), indicating the degree of membership of a given set (that is, the set of not meeting beneficiary demands) and allowing for more nuance in the comparisons. This method can also identify whether or not the same outcome is related to different sets of conditions (recipes), such as if external and internal control factors together relate to satisfying organisational demands, or, for example, to external control and external flexibility.

To compare different conditions (recipes) for positive or negative INGO performance the study proceeded as follows. First, fuzzy scores were calculated for each of the measurements above (beneficiary needs, organisational demands, internal and external focus on control, and internal and external focus on flexibility). This was achieved by computing the relative number of positive or negative statements from the total number of statements per variable in a report. For instance, if a report had 10 statements on organisational demands in total and six were negative and 10 on beneficiary needs but only one was negative, this would be translated into fuzzy scores of 0.6 and 0.1, respectively. They reflect the observation that, according to the evaluator, the case belongs more to the set of projects with negative performance as in not meeting organisational demands than to the set of projects with negative performance as in meeting beneficiary needs. Equal weight is accorded to each negative and positive statement. Since the number of positive, negative, and neutral statements varies, fuzzy score values for negative and positive aspects may not be symmetrical for a given case.

Second, an ‘overall performance’ variable was calculated using the logical AND, as well as the variables (positive or negative) beneficiary needs and organisational demands. The logical AND indicates set intersection (Ragin, 2008). In the case of fuzzy‐set scores, this is calculated with the ‘minimum criterion'. Hence, in the analysis, ‘overall performance', positive or negative, reflects the minimum fuzzy score for performance in each report for realising beneficiaries needs and organisational demands. More intuitively, ‘overall performance’ yields a high value if both measurements of performance have a high value (fuzzy score), reflecting the idea that INGO performance is the result of meeting beneficiary needs *and* meeting organisational demands.

Third, the conditions for positive and negative performance were analysed separately because positive performance might not be equivalent to ‘absence of negative performance'. This implies that the non‐appearance of a combination of conditions leading to good performance will not necessarily lead to negative performance or, vice versa. For this reason, the study opted for a double analysis: one using positive statements across all (outcome and explanatory) conditions, and another using negative statements.

Lastly, the fsQCA reveals which (combination of) conditions (recipes), according to evaluators, consistently relate to positive or negative performance in their reports (meeting beneficiary needs, organisational demands, or both). Using specialised software (Thiem, 2016), the study derived (parsimonious) truth table solutions for positive and negative statements regarding meeting beneficiary needs and organisational demands and overall performance. One should note that, as Baumgartner and Thiem (2015) show, the parsimonious solution generates the most robust QCA solution.

This analytical strategy ensures that as much information as possible is captured from each report, and that the results take into account general patterns in the data and yield comparative information on positive and negative performance, as stated by evaluators.

## Results: general patterns

The results reveal that positive and negative performance (in terms of beneficiary needs, organisational demands, or both) is related to different combinations of control and flexibility conditions (see Tables 1 and 2).

**Table 1:** Recipes for good (positive) performance

	Positive performance		
	Meeting beneficiary needs	Meeting organisational demands	Overall
Solution	1	2	3
Recipes	1.1	2.1	3.1
Internal control (positive)	–	–	–
External control (positive)	–	–	–
Internal flexibility (positive)	–	Present	Present
External flexibility (positive)	Present	–	–
Consistency	0.92	0.98	0.65
Coverage	0.86	0.62	0.99
Cases with consististency ≥ 0.5	Oxfam: Bangladesh, 2005; and Vietnam (Ketsana), 2011. CARE: Haiti, 2005; Pakistan, 2005, 2010; Sri Lanka, 2007; Myanmar, 2008; and Nicaragua, 2008.	CARE: Haiti, 2005.	CARE: Haiti, 2005.

**Source**: authors.

**Table 2:** Recipes for bad (negative) performance

	Negative performance			
	Meeting beneficiary needs	Meeting organisational demands		Overall
Solution	1	2		3
Recipes	1.1	2.1	2.2	3.1
Internal control (negative)	–	Present	–	–
External control (negative)	–	Absent	–	–
Internal flexibility (negative)	–	–	–	–
External flexibility (negative)	**Present**	–	**Present**	**Present**
Consistency	0.91	0.78		0.78
Coverage	0.75	0.99		0.78
Cases with consististency ≥ 0.5	Oxfam: Jamaica, 2008. CARE: Bangladesh, 2008.	CARE: Bangladesh, 2008; and Myanmar, 2008.	Oxfam: Jamaica, 2008. CARE: Bangladesh, 2008.	Oxfam: Jamaica, 2008. CARE: Bangladesh, 2008.

**Source**: authors.

### Recipes for positive performance

Table 1 summarises the analysis of positive performance. It shows the identified combinations of conditions for performance (recipes), as well as measures of consistency (the extent to which a recipe can be considered to be a subset of positive/negative performance) and coverage (a measure of how many relevant cases are accounted for by a given recipe), and provides an indication of exemplary cases for each recipe.^24^

Regarding *positive performance as in meeting beneficiary needs*, it was anticipated that positive evaluations of the organisations’ (internal and external) flexibility in particular would be important. As derived from evaluators’ reports, one parsimonious recipe (1.1 in Table 1) was found, relating to having positive evaluations of external flexibility—that is, having good coordination and communication with local actors, as well as community participation and capacity‐building at the local level. This resonates with the study expectations.

Regarding *positive performance as in meeting organisational demands*, it was anticipated that (internal and external) control dimensions would be important. One recipe (2.1 in Table 1) was identified that indicated that organisational demands were related to positive evaluations of internal flexibility in the reports. This is contrary to expectations—a point to which the paper returns in the conclusion.

The parsimonious solution for overall *positive* performance, as in meeting beneficiary needs *and* organisational demands, is also related to the presence of positive statements on internal flexibility in the reports (see recipe 3.1 in Table 1). Hence, in the sample of evaluation reports, having well‐trained and knowledgeable staff members who can be deployed quickly is associated with meeting both beneficiary needs and organisational demands.

The exemplary cases in the sample (those having a case consistency score of more than 0.5) pertaining to recipe 1.1 can be found within CARE and Oxfam, denoting that there are no fundamental differences between the two organisations linked to meeting beneficiary needs. The exemplary case for recipe 2.1 (organisational demands) is the report on CARE's emergency response in Haiti (2005).

### Recipes for negative performance

Table 2 summarises the recipes for negative performance.

Regarding *negative performance as in not meeting beneficiaries’ needs*, a negative evaluation of external flexibility dimensions (such as not aligning with local partners and communities) was found to relate to negative performance (recipe 1.1. in Table 2). This is according to expectations, and a reverse image of the result for positive performance. Once again, exemplary cases can be found within CARE and Oxfam.

Regarding *negative performance as in not meeting organisational demands*, negative performance in meeting organisational demands was found to relate to two recipes. One recipe (2.2 in Table 2) shows a relation between negative performance concerning organisational demands and not performing well with respect to external flexibility factors in the view of evaluators. Consequently, lacking external flexibility pertains to problems in meeting both beneficiary needs and organisational demands, which is also represented in the recipe for overall negative performance (3.1 in Table 2).

The second recipe of conditions connected to not achieving organisational demands (2.1 in Table 2) is composed of negative assessments of internal control factors (planning, financial and information management, strategy, and standards), which resonates with the CVF. Here, the latter are found in combination with the absence of negative statements on external control (PR and accountability), implying that organisational demands were not fulfilled, even though negative statements on external control were absent.

The above results suggest that to achieve positive performance in meeting organisational demands, it is important not only to do well with regard to internal flexibility dimensions (see Table 1), but also not to perform badly in relation to internal control dimensions (see Table 2). This infers that the mirror image of positive performance vis‐à‐vis organisational demands is not the same for negative performance.

## Results: inductive analysis

To interpret the results further, the study returned to the cases. It focused on the report on CARE's emergency response in Haiti (2005), since this is exemplary of all recipes of positive performance, both in terms of meeting beneficiary needs and organisational demands. In addition, the report on CARE's activities in Bangladesh (2008) is discussed, as it is exemplary for the recipe of negative performance.

### Haiti, 2005: the importance of external and internal flexibility

Apropos of meeting beneficiary needs, evaluators praised CARE for the appropriateness of its aid to the population in terms of water supply, sanitation, and food and cash for work activities. With regard to coverage, the evaluators judged that CARE did better than other organisations in reaching groups in need. This was associated with good performance along the external flexibility dimensions of local capacity‐building, community participation, and external communication and coordination with local actors. CARE was praised for providing financial and logistical support to governmental partners, especially for capacity‐building efforts during a school project, deemed to have reinforced the partnership with local authorities. The organisation was also applauded for its efforts to mobilise and revitalise existing community groups; for instance, CARE facilitated the initiation of clean‐up brigades, which removed mud and rubbish left by the storm from towns. As for external communication with local actors, the following conclusion was reached: ‘CARE Haiti's record in coordinating with other stakeholders on the ground was admirable’ (*Independent Evaluation of CARE's Humanitarian Response to Flooding Resulting from Tropical Storm Jeanne in Haiti (North‐west and Artibonite Provinces)*, 2005, p. 12).

Regarding meeting organisational demands in Haiti, the evaluators stated that CARE helped to prevent malnutrition and the transmission of waterborne and other diseases, and that it was effective in responding to immediate needs (food and water) and in facilitating the rebuilding of a school. Here, this relates to performing well in terms of internal flexibility through the presence of well‐trained and knowledgeable staff. Evaluators were especially positive about the quick deployment of personnel within Haiti and from abroad. This was facilitated by various regional CARE structures, such as the Global Emergency Group, and the swift recovery of local staff after the storm. Furthermore, the local knowledge of staff was praised: ‘CARE's strength in Gonaïves in September 2004 was its local knowledge, contacts, pre‐existing protocols with local government institutions’ (*Independent Evaluation of CARE's Humanitarian Response to Flooding Resulting from Tropical Storm Jeanne in Haiti (North‐west and Artibonite Provinces)*, 2005, p. 25).

### Bangladesh, 2008: external flexibility and internal control

Problems in the area of external flexibility—related to community participation, capacity‐building, and coordination with local actors—hindered the ability of the CARE Bangladesh team to perform well in meeting both beneficiary needs and organisational demands. The evaluators mention that CARE failed to include local communities in all phases of interventions, such as in needs assessment, project proposal development, and aid distribution processes. This led, inter alia, to the use of a certain type of cladding for shelter that was deemed unpractical by beneficiaries, the unannounced placement of a water plant in a community, poorly functioning water management committees, and changes to boat‐building procedures without consultation with local fishers. Moreover, aid recipients were not informed about the contents of aid packages, and since these varied substantially (which can be regarded as a problem of coherence), recipients became suspicious about whether packages had been altered by the distributing local NGO. What is more, beneficiaries were not informed if they had been selected for specific aid distributions. CARE also missed opportunities for capacity‐building: it did not manage to help and train its local partners and to develop local capacity for water management.

Regarding external communication and coordination with local actors, the evaluators especially discussed the relationship between CARE Bangladesh and its local partner organisations (*Independent Evaluation of CARE‐B's Cyclone Sidr Response Program*, 2008, p. 19):

*the relationship between CARE‐B [CARE Bangladesh] and the PNGOs [partner NGOs] were client–contractor rather than partnerships as PNGOs were very restricted in what they could do and were not consulted on the design or implementation of activities. PNGOs were expected to implement a defined task within a specified time period and budget. Even key items in the budgets were fixed by CARE‐B and did not take into account actual market prices or availability of products*.

In addition, CARE did not inform them about changes to plans or projects, so that the partners had no opportunity to integrate local knowledge of the appropriateness of these alterations. Partner organisations claimed that, because of CARE's rigid attitude, they could not deliver. The evaluators attributed this to CARE Bangladesh's lack of experience in working with partners in emergency relief contexts.

The Bangladesh case is also exemplary for a lack of performance with regard to organisational demands, relating to problems with internal control, next to external flexibility issues. Examples are inadequate aid packages, delays in the delivery of World Food Programme aid packages, and finalising the design of latrines, as well as technical problems with hand tube‐wells and the supply of sanitary napkins without issuing communication on their use. The evaluators reported problems in all four internal control domains, of which just a few are cited here. As for financial management, there were delays in payments and inconsistencies in financial reporting. In terms of information management, there was no skilled staff to execute tasks. In addition, opportunities were missed to collect baseline information on households that later would receive aid. Concerning planning, there were delays in recruiting senior personnel for the emergency response. Regarding strategy and standards, CARE Bangladesh did not have an updated emergency plan and did not always meet Sphere standards, such as for non‐food and food item packages.

### Comparing reports

Given that this study is based on the frequency of negative and positive statements that all have equal weight, singular but crucial statements in the reports might be overlooked. When coding the reports, attention was paid to these statements and two issues were inductively identified. First, three CARE reports and one Oxfam report mentioned tensions between development aid and emergency aid staff and orientations, especially if the two types of personnel were not integrated. This impaired smooth coordination and cooperation in the teams. This issue was identified in the CARE reports on Cyclone Nargis in Myanmar (2008), the tsunami in Sri Lanka (2005–07), and Tropical Storm Jeanne in Haiti (2005), and the Oxfam report on Jamaica (2006). The *Evaluation of CARE Myanmar's Cyclone Nargis Response* (2008, p. 20) illustrates this point:

*There were clear difficulties in terms of relationships between the development staff and the emergency staff, old staff and new staff, field staff and HQ staff etc. These difficulties included gaps in terms of understanding of roles and responsibilities, differences of opinion about how to approach the issue of timeliness, different understandings of systems and structures, differences regarding the approach to quality programming etc*.

Second, evaluators repeatedly stated in two Oxfam reports that donor demands led the organisation to make adjustments to activities at the expense of the appropriateness of aid, thereby compromising beneficiary needs. These comments pertained to the Bangladesh (2005) and the Jamaica (2006) reports, the latter being exemplary for negative performance in meeting beneficiary needs and organisational demands. A similar remark was made in the *Independent Evaluation of CARE‐B's Cyclone Sidr Response Program* (2008, p. 9):

*The Oxfam flood response was donor based and it was designed with donor money in mind, and this can detract from a needs based approach. This approach has influenced the number of families to be covered in a district regardless of the severity of the disaster. Both in restricting the numbers in some areas and spreading the response too thinly in others*.

The same report (p. 16) also mentioned the following donor pressures:

*The factor that was missing in strategy development was the requirement of affected communities to be involved in all stages of the planning, design and implementation of humanitarian actions. Meeting this requirement is challenging as donors are often prescriptive about what they are willing to fund and want proposals to be specific about items to be provided or number of packages to be distributed. This leaves limited scope for working with communities over time to determine what they need. For example, DFID contacted CARE‐B about their interest in a WATSAN project*.

## Discussion

This paper has explored organisational conditions related to the positive and negative performance of humanitarian INGOs in relation to disasters triggered by natural hazards, and assessed whether they can meet beneficiary needs and organisational demands simultaneously. In so doing it analysed 11 evaluation reports on humanitarian aid projects after disasters triggered by natural hazards, implemented by CARE and Oxfam, and used this expert information for fsQCA.

The study found, first, that the CVF was useful in appraising performance of humanitarian aid projects in this particular sample of reports. As expected, meeting beneficiary needs was associated with good performance in terms of external flexibility aspects. Unexpectedly, internal flexibility was also important in fulfilling organisational demands (instead of internal or external control). This finding relates to discussions about ‘structured flexibility', suggesting that structures and procedures (and thus control) might be necessary to facilitate flexibility and manage potential tensions deriving from competing demands (Cameron and Levine, 2006; Battilana et al., 2015; Smith and Tracy, 2016). Negative statements about external flexibility dimensions—that is, failing in establishing external relations with local actors—were connected to not satisfying beneficiary needs *and* organisational demands. This hints at the importance of humanitarian INGOs having flexible organisational arrangements and proactive linkages with local actors.

Second, in the case of meeting organisational demands, evaluator statements about positive performance were not the exact mirror image of evaluator statements about negative performance in the sample. Whereas performing well in terms of internal flexibility was important for meeting organisational demands, the negative evaluation of internal control dimensions related to *failing* to meet organisational demands.

Third, the results for organisational demands suggest that INGOs should not only *perform* well in some areas, but also should not *fail* in others: according to evaluators, performing well with regard to internal flexibility was important in fulfilling these demands, whereas performing badly with respect to internal control was related to failure. More generally, the analysis confirms that humanitarian aid organisations need to have capabilities to manage several internal and external control and flexibility dimensions simultaneously in their endeavour to realise multiple demands (Besharov and Smith, 2014; Smith and Lewis, 2014).

Fourth, reports contained negative and positive statements about performance and external/internal flexibility and control dimensions. Hypothetically, reports could be an exemplary case of positive and negative performance. Interestingly, eight reports are exemplary cases in the positive performance category and three reports are exemplary cases in the negative performance category. It is noteworthy that the exemplary cases of positive and negative performance in the various recipes consisted of a mixture of Oxfam and CARE reports, indicating similarities in the projects of these two INGOs that perform both well and less well.

Lastly, through inductive analysis, the study identified some potential complementary explanations for good and bad performance. These pertained to donor pressures that led organisations to adapt their aid activities at the expense of beneficiaries and clashes between development aid and emergency aid staff and orientations, a well‐known source of tension in humanitarian aid provision (Buchanan‐Smith and Maxwell, 1994; Audet, 2015).

## Conclusion

This paper is a first step in appraising the performance of INGOs in humanitarian aid projects on the basis of evaluation reports. It is acknowledged that evaluation reports are not written for the purpose of academic research and that the results of the investigation should be seen in the light of this fact. For instance, some factors in which the authors are theoretically interested were not discussed in the reports as extensively as hoped. The decision was taken, therefore, to exclude these reports from the examination for the purpose of validity. Hence not all of the reports are suitable for this type of analysis. Conversely, all relevant dimensions might not have been included in the coding. For example, with regard to external flexibility, the presence of external pressures that led to aid diversion or some groups being favoured over others were not specifically coded; it would be interesting to include such a dimension in a future study. In addition, the possibilities for inductive, exploratory scrutiny were limited to the pages of text written by the evaluator.

The QCA analysis was based on the relative frequencies of positive and negative evaluation statements about the performance of two quite similar INGOs providing aid in a comparable time frame and disaster setting. Future research might further expand the number of reports in the sample to reach saturation in the ‘performance recipes'. One option would be to include more evaluation reports of the same organisations, initially on the same type of crisis. If saturation of recipes for (in)adequate performance in both organisations in environments characterised by disasters triggered by natural hazards is achieved, one could opt to add reports of other types of emergencies to the sample, such as armed conflicts. In a third step, one could add reports of other not so similar INGOs, so as to be able to compare evaluated performance across INGOs. Such an inquiry could contribute to the development of hypotheses on explanations of differences in performance between INGOs.

Another avenue for future research pertains to the inductive observation that some cases seemed to show unresolved tensions between an orientation towards development aid and emergency aid. This study was not able to pinpoint why this was a problem in these particular cases and not in others. Consequently, it is not really known why the tension hampered performance in some projects and was absent or, if present, managed successfully, in other cases. This matter could be resolved by using additional data, such as interview information, or by considering other types of documents to acquire more in‐depth information on the organisational context.

Nevertheless, evaluation reports on INGO performance in humanitarian crises are a valuable source of information for exploring which organisational conditions—albeit in the eyes of evaluators—relate to performance. The analyses presented in this paper imply that project performance in this sector reflects a balancing act as INGOs learn to succeed in some areas while trying not to fail in others.

## Annexe

### Coding

∗ Taken directly from the HG code book.
∗∗ Combination of codes in the HG code book.
∗∗∗ Adapted code from the HG code book.

### Outcomes

To gauge whether beneficiary needs and organisational demands had been met, the authors coded neutral, positive, and negative statements in the evaluation reports using the well‐known and widely employed OECD–DAC criteria.^25^ A statement was coded as negative if the text refers to an action that occurred, or did not occur, and that had a negative effect, whereas a statement was coded as positive if the text refers to an action that occurred had a positive effect. See Table A1.

**Table A1:** Outcomes

Outcomes	Related OECD–DAC criteria	Definition and online sources	Codes
Beneficiary needs	Appropriateness beneficiaries∗	The extent to which the aid activity fits the priorities and policies of the target group or recipients. Sources: https://www.alnap.org/system/files/content/resource/files/main/eha-2006.pdf; and http://www.netpublikationer.dk/um/7571/html/chapter05.htm (last accessed on 21 April 2020).	Appropriateness beneficiaries positive Appropriateness beneficiaries negative Appropriateness beneficiaries neutral
	Coverage∗	The question of who was supported and reached by humanitarian action, and who was not. Source: adapted from https://www.alnap.org/system/files/content/resource/files/main/eha-2006.pdf (last accessed on 21 April 2020).	Coverage positive Coverage negative Coverage neutral
Organisational demands	Coherence∗	The extent to which policies (humanitarian, developmental, trade, and military) are consistent, and that all policies take into account humanitarian and human rights considerations. Also related to policy, plans, and procedures. Source: https://www.alnap.org/system/files/content/resource/files/main/eha-2006.pdf (last accessed on 21 April 2020).	Coherence positive Coherence negative Coherence neutral
	Effectiveness∗∗	The extent to which the activity/programme achieves its purpose or objectives (also in terms of timeliness and cost effectiveness). Sources: https://www.alnap.org/system/files/content/resource/files/main/eha-2006.pdf (last accessed on 21 April 2020); see also Chianca (2007).	Effectiveness positive Effectiveness negative Effectiveness neutral

**Source**: authors.

### Flexibility conditions

To determine whether flexibility conditions were met, the authors coded neutral, positive, and negative statements in the evaluation reports on the following aspects shown in Table A2.

**Table A2:** Flexibility conditions

Organisational conditions	Sub‐ncomponents	Code categories with definition	Codes
Flexibility conditions	Internal focus on flexibility	*Quickly available and deployable staff*∗ The swift deployment of experienced coordination experts and other specialised humanitarian personnel. Source: https://www.unocha.org/our-work/coordination/surge-capacity (last accessed on 21 April 2020).	HRM – deployment staff pos HRM – deployment staff neg HRM – deployment staff neutral
		*Experiences and knowledgeable staff*∗∗ The experience and skills that one has in doing a particular job. Also related to knowledge of a local situation. Source: based on http://www.macmillandictionary.com/dictionary/british/work-experience (last accessed on 21 April 2020).	HRM – experience and knowledge of staff pos HRM – experience and knowledge of staff neg HRM – experience and knowledge staff neutral
		*Training of staff*∗ The systematic acquisition of skills, rules, concepts, or attitudes. Source: Patterson et al. (2010, p. 295).	HRM – training of staff pos HRM – training of staff neg HRM – training of staff neutral
	External focus on flexibility	*Local capacity‐building*∗ The process by which individuals, organisations, institutions, and societies develop abilities to perform functions, solve problems, and set and achieve goals. Ideally it should be based on an understanding of the possibilities and limitations of the environment and of the needs perceived by the target group. Source: adapted from http://www.undp.org/content/dam/aplaws/publication/en/publications/capacity-development/capacity-development-a-undp-primer/CDG_PrimerReport_final_web.pdf (last accessed on 21 April 2020).	Capacity building pos Capacity building neg Capacity building neutral
		*Community participation*∗ Key stakeholders (and especially the proposed beneficiaries) of a policy or intervention are closely involved in the process of identifying problems and priorities and have considerable control over analysis and the planning, implementation, and monitoring of solutions. Source: based on Beck et al. (2003).	Community participation pos Community participation neg Community participation neutral
		*External communication and coordination with local actors*∗∗∗ Issues concerning communication and coordination between individuals/units of the organisation under evaluation and any other local actor, such as the government, other NGOs, and the community. Source: based on UN OCHA (2012).	External comm and coord local actor pos External comm and coord local actor neg External comm and coord local actor neutral

**Source**: authors.

### Control conditions

To assess whether control conditions were met, the authors coded neutral, positive, and negative statements in the evaluation reports on the following aspects shown in Table A3.

**Table A3:** Control conditions

Organisational conditions	Sub‐components	Code categories with definition	Codes
Control conditions	Internal focus on control	*Planning of the project/activities*∗ The process of making plans for something; the planning stage of the operation. Source: http://oxforddictionaries.com/definition/english/planning?q=planning (last accessed on 21 April 2020).	Planning pos Planning neg Planning neutral
		*Strategy∗∗ & meeting standards*∗∗∗ Strategy deals mainly with actions; it refers to the methodology used to achieve the targets that are prescribed by the policy. Also includes statements about policies. Source: adapted from http://www.managementstudyguide.com/business-policy.htm (last accessed on 14 April 2020).	Strategy and standards pos Strategy and standards neg Strategy and standards neutral
		*Information management*∗ How an organisation uses, spreads, stores, and applies information within the organisation and/or programme. Source: https://www.unocha.org/our-work/information-management (last accessed on 21 April 2020).	Information management pos Information management neg Information management neutral
		*Financial management*∗∗ Planning, organising, controlling, and reporting on the financial resources to achieve organisational goals. Also related to funding and resource mobilisation. Sources: http://www.fritzinstitute.org/prsrm-HLS.htm; and http://oxforddictionaries.com/definition/english/procurement?q=Procurement (last accessed on 21 April 2020).	Financial management pos Financial management neg Financial management neutral
	External focus on control	*PR and accountability activities*∗∗ Press and media mentioning, representation of the organisation, and reputation of the organisation. Key questions include: is the organisation widely known? and how is the organisation perceived by others? Any mechanism/process via which the organisation can account for its activities. Accountability is a process of taking account of, and being held accountable by, different stakeholders, primarily those who are affected by the exercise of power. Source: Humanitarian Accountability Partnership (2011, p. 6).	PR and accountability pos PR and accountability neg PR and accountability neutral
		*External communication and coordination with donors*∗∗∗ Issues regarding communication and coordination between individuals/units of the organisation under evaluation and any donor. Source: based on Roberts, Jones, III, and Fröhling (2005).	External comm and coord donors pos External comm and coord donors neg External comm and coord donors neutral

**Source**: authors.

## Acknowledgements

The data for this paper was partly generated through the HG database which was funded by a grant from the Humanitarian Innovation Fund. This study is part of the research program Sustainable Cooperation – Roadmaps to Resilient Societies (SCOOP). The authors are grateful to the Netherlands Organization for Scientific Research (NWO) and the Dutch Ministry of Education, Culture and Science (OCW) for generously funding this research in the context of its 2017 Gravitation Program (grant number 024.003.025).

## Endnotes

1 See, for example, the reports about fraud in one of Amnesty International's offices (Chingono, 2019) and about sexual abuse within Oxfam (Ratcliffe, 2019).

2 With the exception of Oxfam's *Evaluation of the Bangladesh Emergency Flood Response*. The decision was taken to retain this report, though, because of a fair balance of negative and positive statements about project performance.

3 For more information, see https://www.rug.nl/research/globalisation-studies-groningen/projects/genomeproject (last accessed on 9 April 2020).

4 This selection took place in 2016–17.

5 For more information, see https://www.alnap.org/help-library/independent-evaluation-of-cares-humanitarian-response-to-flooding-resulting-from (last accessed on 21 April 2020).

6 For more information, see https://www.alnap.org/help-library/independent-evaluation-of-care-internationals-earthquake-response-in-northern-pakistan (last accessed on 21 April 2020).

7 For more information, see https://www.careevaluations.org/wp-content/uploads/evaluations/ken-emergency-respnse-erdap-final-jan-08.pdf (last accessed on 21 April 2020).

8 For more information, see https://www.alnap.org/help-library/final-evaluation-of-care-australia-supported-tsunami-response-in-trincomalee-and (last accessed on 21 April 2020).

9 For more information, see http://www.careevaluations.org/wp-content/uploads/evaluations/uganda-flood-response-evaluation-evaluation-2008-february-doc.pdf (last accessed on 21 April 2020).

10 For more information, see https://www.alnap.org/help-library/independent-evaluation-of-care-bangladeshs-cyclone-sidr-response-program (last accessed on 21 April 2020).

11 For more information, see https://www.alnap.org/help-library/evaluation-of-care-myanmars-cyclone-nargis-response (last accessed on 21 April 2020).

12 Document no longer available online as of 23 April 2020. Copy on file with the authors.

13 For more information, see https://www.dec.org.uk/sites/default/files/PDFS/final-report-care-sindh-evaluation-0811.pdf (last accessed on 21 April 2020).

14 For more information, see https://www.alnap.org/help-library/evaluation-of-the-bangladesh-emergency-flood-response (last accessed on 21 April 2020).

15 For more information, see https://oxfamilibrary.openrepository.com/handle/10546/119442 (last accessed on 21 April 2020).

16 For more information, see https://oxfamilibrary.openrepository.com/bitstream/handle/10546/119439/er-flood-response-georgia-010106-tor-en.pdf?sequence=3 (last accessed on 21 April 2020).

17 For more information, see https://www.alnap.org/help-library/evaluation-of-the-response-to-hurricane-dean-in-jamaica-st-lucia-and-dominica (last accessed on 21 April 2020).

18 For more information, see https://www.alnap.org/help-library/evaluation-report-typhoon-ketsana-emergency-response-project-in-kon-tum-province-rvna79 (last accessed on 21 April 2020).

19 For more information, see https://www.elrha.org/project-blog/humanitarian-genome-knowledge-management-tool-available-use/ (last accessed on 9 April 2020).

20 For more information, see https://www.elrha.org/wp-content/uploads/2017/08/hif-alnap-groningen-humanitarian-genome-2014.pdf (last accessed on 21 April 2020).

21 See also the ALNAP blog on ‘DAC criteria for evaluating development assistance’ at https://www.alnap.org/help-library/dac-criteria-for-evaluating-development-assistance (last accessed on 9 April 2020) and the annexe for the extensive coding scheme, definitions, and related sources.

22 The paper addressed earlier the risk of diversion of aid and of favouring one group over another. If this was referred to in the reports, it was coded in terms of negative statements on ‘appropriateness beneficiaries’ or ‘coverage', depending on the content of the statement.

23 The paper refers above to the risk of aid diversion or favouring one group over another as part of external dynamics. Endnote 22 explains how this would be reflected in negative statements on meeting beneficiary demands.

24 Consistency and coverage scores range between zero and one; the higher these scores, the better.

25 For more information on these criteria, see Beck (2006).
